# Habitual Combined Exercise Protects against Age-Associated Decline in Vascular Function and Lipid Profiles in Elderly Postmenopausal Women

**DOI:** 10.3390/ijerph17113893

**Published:** 2020-05-30

**Authors:** Elizabeth J. Pekas, John Shin, Won-Mok Son, Ronald J. Headid, Song-Young Park

**Affiliations:** 1 School of Health and Kinesiology, University of Nebraska at Omaha, Omaha, NE 68182, USA; lizpekas@unomaha.edu (E.J.P.); jshin1128@gmail.com (J.S.); wson@unomaha.edu (W.-M.S.); rheadid@unomaha.edu (R.J.H.III); 2 Wiess School of Natural Sciences, Rice University, Houston, TX 77005, USA

**Keywords:** cardiovascular disease, metabolic syndrome, sarcopenia, vascular dysfunction, walking distance

## Abstract

Postmenopausal status is associated with increased risks for cardiovascular diseases (CVD). This study investigated differences in vascular function, lipids, body composition, and physical fitness in elderly postmenopausal women active in combined resistance and aerobic exercise (CRAE) training for 1 year versus a sedentary cohort of similar-in-age counterparts. Elderly postmenopausal women performing habitual CRAE training for 1 year (age ~75 year; CRAE, *n* = 57) and elderly sedentary postmenopausal women (age ~78 year; SED, *n* = 44) were recruited. Arterial stiffness (brachial-to-ankle pulse-wave velocity, baPWV), blood pressure, blood lipids, anthropometrics, 2-min walking distance, and muscular strength were assessed for both groups. There were significant differences for baPWV, systolic blood pressure, low-density lipoprotein, and body fat percentage, which were significantly lower (*p* < 0.05) in CRAE vs. SED, and both 2 min walking distance and muscular strength were significantly greater (*p* < 0.05) in CRAE vs. SED. These results indicate that elderly postmenopausal women participating in habitual CRAE training may have better protection against risks for CVD and have better physical fitness compared to SED counterparts.

## 1. Introduction

Age is a well-known non-modifiable risk factor for cardiovascular disease (CVD) [1] which is commonly accompanied by increased abdominal obesity, triglyceride levels, blood pressure (BP), glucose levels, decreased high-density lipoprotein (HDL) cholesterol, and increased prevalence metabolic syndrome [2]. It has been projected that by the year 2030, nearly 25% of the US population will be 66 years of age and older [3]. Menopause is considered a natural part of aging and is associated with a greater risk for metabolic syndrome as well as increased levels of arterial stiffness, BP, blood lipids, body fat accumulation, reduced resting energy expenditure, and increased prevalence of sarcopenia [4,5,6,7]. Importantly, several of these risk factors for atherosclerotic diseases are considered to be silent and often show no symptoms [8]; in particular, vascular dysfunction is accelerated after menopause [9]. Endothelial dysfunction and arterial stiffness are thought to precede CVD manifestation and have been reported to be independent predictors of cardiovascular event occurrence [10,11]. This vascular dysfunction and other risk factors, such as elevated BP, are thought to be partially attributed to estrogen deficiency, which may therefore put postmenopausal women at a higher risk for CVD manifestation [9,12]. These risk factors, if left alone, could contribute to a lower quality of life and ultimately increase the potential of CVD development and mortality in postmenopausal women.

We have previously shown that combined resistance and aerobic exercise (CRAE) training is effective for improving several risk factors for CVD and metabolic syndrome in postmenopausal women and adolescents, including reductions in BP, arterial stiffness, and improvements in nitric oxide (NO) bioavailability, insulin sensitivity, and aging-related hormones such as estrogen, thereby potentially decreasing risks for CVD development [13,14,15,16,17,18]. These programs consisted of CRAE training sessions that were 50–60 min, 3–5 times per week, over a time period of 12 weeks [13,14,15,16]. Although these exercise programs produced beneficial results after only 12 weeks, it has been suggested that habitual exercise is fundamental for promoting cardiovascular health and reduced disease risks in populations of all ages and conditions. Aerobic exercise training for 1 year (≥45 min of moderate-to-vigorous intensity exercise ~3–4 times per week) has been shown to significantly reduce adiposity levels in previously sedentary postmenopausal women [19], and yearlong resistance training programs for postmenopausal women (3 times per week for 60–75 min per day, 70–80% 1RM intensity focusing on large muscle groups) have demonstrated significant improvements in muscular strength and lean mass [20]. Additionally, 1 year of combined aerobic and resistance exercise training showed improvements in insulin resistance, body fat percentage, and skeletal muscle mass in postmenopausal women [21]. However, the impacts of habitual CRAE training over 1 year on risk factors for CVD in postmenopausal women compared to their sedentary counterparts have not been previously investigated. The aim of this study was to examine the arterial stiffness, BP, blood lipid profiles, body composition, resting metabolic rate (RMR), walking capacity, and muscular strength in elderly postmenopausal women regularly participating in CRAE training for at least 1 year in comparison to a sedentary cohort of similar age. We hypothesized that habitual CRAE training group would demonstrate healthier levels of arterial stiffness, BP, blood lipid profiles, body fat percentage, RMR, walking capacity, and muscular strength compared to the sedentary group.

## 2. Materials and Methods

### 2.1. Participants

Participants (age CRAE: ~75 year, SED: ~78 year) were recruited at multiple community health centers using flyers and referrals. All participants were postmenopausal (no menses for at least 12 consecutive months) and were considered mid-to-late elderly [22]. Participants were either classified as habitually participating in CRAE training for 1 year or were sedentary (no participation in regular exercise within the previous year). The CRAE group participated in group exercise training classes at the community fitness centers and performed CRAE training programs at least 3 times per week over the course of 1 year as previously described (Table 1) [13,14,15,16]. The exercise sessions were supervised and administered by qualified trainers. The SED group was recruited from the same community center but registered in other activities (i.e., painting and floral design classes) and did not regularly engage in exercise training. Exclusion criteria included current smoker, cardiovascular diseases, renal diseases, thyroid diseases, hormone replacement therapy, use of weight loss diet within the previous year, and/or psychiatric conditions or cognitive dysfunction. All protocols were performed in accordance with the Declaration of Helsinki. The IRB in South Korea was not initiated until 2013, and therefore an IRB number was not required to carry out this study (performed in 2010). Written, informed consent was received form each participant despite no IRB approval requirements for study initiation, therefore there were no ethical violations or other issues for the study.

### 2.2. Study Design

This study used a retrospective cohort study design and used convenience sampling. The enrollment of participants was performed by a research coordinator who was not involved in the laboratory assessments. The research coordinator who was not involved in laboratory assessments evaluated the inclusion criteria to determine if participants would be placed in the CRAE training group (CRAE), sedentary control group (SED), or would be ineligible for the measurements in this study. The research coordinator determined eligibility and group placement using a survey about participant exercise participation, health history (see exclusion criteria in Section 2.1), and medication use. If participants were deemed eligible according to the eligibility criteria, participants were grouped by their exercise participation history (i.e., CRAE group or SED group). Arterial stiffness, BP, blood lipid panels, anthropometrics, RMR, walking capacity, and muscular strength were evaluated for both groups. All participants were asked to abstain from all current medications, alcohol, caffeine, and physical activity for a minimum of 24 h prior to the measurements. Measurements for all participants were performed at the same time of day after an overnight fast (8:00 a.m., ±1 h). The lab personnel performing the measurements were blinded to the participant allocation.

### 2.3. Blood Sampling and Analysis

Blood samples (10 mL) were obtained at 8:00 a.m. ± 1 h after an overnight fast. Samples were collected by a trained phlebotomist from an antecubital vein in with serum separator tubes (Becton Dickinson, Franklin Lanes, NJ, USA). Samples were centrifuged at 1500× *g* for 10 min. Serum samples were extracted and stored in polypropylene vials at −20 °C for later analysis. Serum total cholesterol (TC), high-density lipoprotein (HDL), low-density lipoprotein (LDL), and triglycerides (TG) were measured with enzymatic assay procedures (Laboratory Corporation of America, Norfolk, VA, USA). Inter-assay coefficients of variation were 2.3% and 2.1% for low and high total cholesterol controls, respectively, and 6.5% on HDL cholesterol control (mean 1.27 mmol/L), which comply with National Cholesterol Education Programme recommendations. Very low-density lipoprotein (VLDL) cholesterol was calculated by [VLDL-C] = [TG]/5, and LDL cholesterol was calculated by [LDL-C] = [T-CHOL] − [HDL-C] − [VLDL-C], where C is cholesterol and TG is triglycerides.

### 2.4. Arterial Stiffness and Blood Pressure

Brachial-to-ankle pulse-wave velocity (baPWV), an index of peripheral arterial stiffness, and BP were assessed with a volume plethysmographic device (VP-1000, Colin Company, Komaki, Aichi, Japan). Electrocardiogram (EKG) electrodes were placed on the forearms, a heart microphone was placed on the left parasternal border of the fourth intercostals space, and BP cuffs with plethysmographic sensors were placed on the brachial and posterior tibial arteries of the left and right side [13]. BP, EKG, and pulse waveforms were recorded simultaneously for 10 **s** and were analyzed as previously described [13].

### 2.5. Anthropometrics

Participant height was measured to the nearest 0.1 cm using a standard stadiometer without shoes. Body mass and body fat percentage were evaluated using bioelectrical impedance analysis (BIA) with the InBody 230 (Biospace, Seoul, South Korea) to the nearest 0.1 kg and 0.1%, respectively [23]. Body mass index (BMI) was calculated as the total body mass divided by height squared (kg/m^2^).

### 2.6. Resting Metabolic Rate and Dietary Intake

RMR was calculated using indirect calorimetry (OxyCon Pro, Viasys Healthcare, Conshohocken, PA, USA). Subjects were asked to rest in the supine position on a padded table. Subjects were informed to breathe normally into the instrument and to not fall asleep during the measurement. Measurements were taken for 30 min [23].

Participants were asked to track their dietary intake and to not change their dietary habits for 1 week after study enrollment. After 1-week, dietary logs were collected to calculate the estimated kcal/day intake for the CRAE and SED groups.

### 2.7. Walking Capacity

Walking capacity was determined using the 2-min walk test. Participants were tested in a quiet uncarpeted hallway where they were asked to walk back and forth between 2 pieces of tape on the floor (50 m apart) and to cover as much distance as possible within 2 min. The distance walked in meters was recorded as the 2-min walking distance score.

### 2.8. Muscular Strength

Lower body muscular strength (quadriceps) was determined using the one repetition maximum (1RM) test using a leg extension machine (Cybex 6000, Lumex, Albertson, NY, USA). The 1RM was measured using the dominant leg only (not double-leg extension), and the 1RM was achieved within 5 or fewer attempts. The heaviest weight lifted using proper form and full range of motion was recorded as the 1RM. Upper body muscular strength (biceps) was determined using the 1RM test using a standard bicep curl machine (Optima Series Biceps Curl, Life Fitness, Rosemont, IL, USA). The 1RM was measured using two-arm bicep curl and was achieved in 5 or fewer attempts. The heaviest weight lifted using the proper form and full range of motion was recorded as the 1RM.

### 2.9. Statistical Analysis

The Shapiro-Wilk test was used to determine the normal distribution of data. Independent *t* tests were used to determine the group differences between the CRAE and SED groups. Data were analyzed with SPSS statistical software (SPSS 24, SPSS Inc., Chicago, IL, USA). Statistical significance was set to *p* < 0.05.

## 3. Results

**Figure 1:**
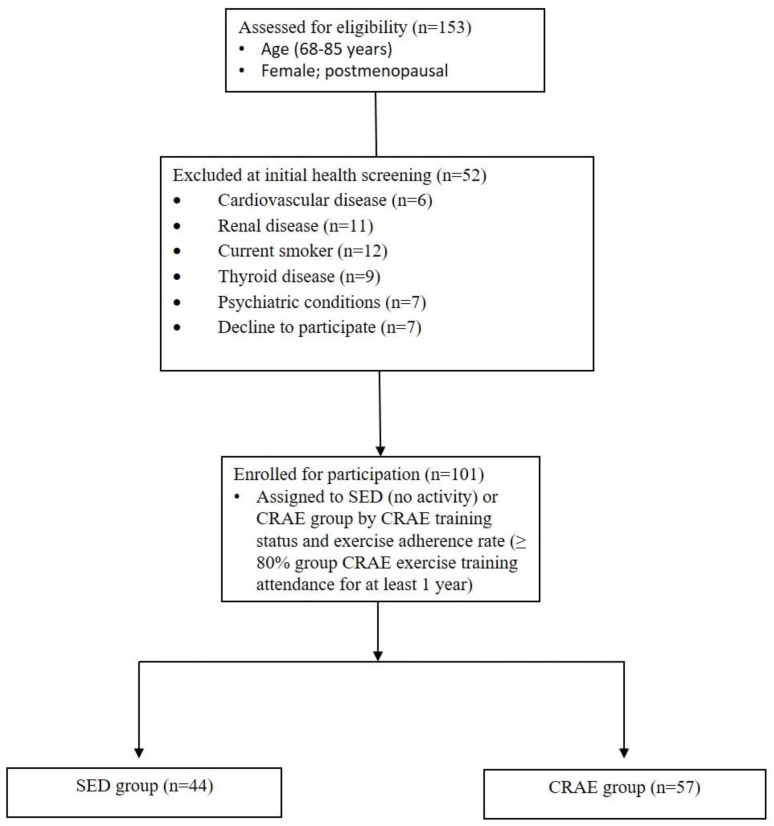
Study subject eligibility, screening, and allocation.

**Figure 2:**
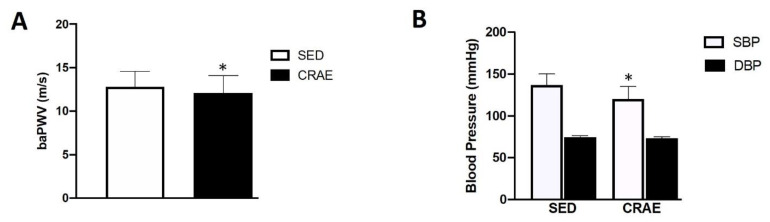
Differences in brachial-to-ankle pulse wave velocity (baPWV) and blood pressure (BP) between the combined resistance and aerobic exercise training (CRAE) and the sedentary (SED) groups. (**A**) The CRAE group had significantly lower baPWV (m/s) compared to the SED group (12.8 ± 1.8 vs. 12.1 ± 2.0 m/s, SED vs. CRAE, respectively; *p* = 0.041) (**B**) The CRAE group had significantly lower systolic BP (SBP) compared to the SED group (137.0 ± 33.0 vs. 120.0 ± 15.0 mmHg, SED vs. CRAE, respectively; *p* = 0.03). There were no differences in diastolic BP (DBP) between groups (68.0 ± 15.0 vs. 67.7 ± 10.0 mmHg, SED vs. CRAE, respectively; *p* = 0.12). Values are Mean ± SD. * *p* < 0.05 vs. SED.

**Figure 3:**
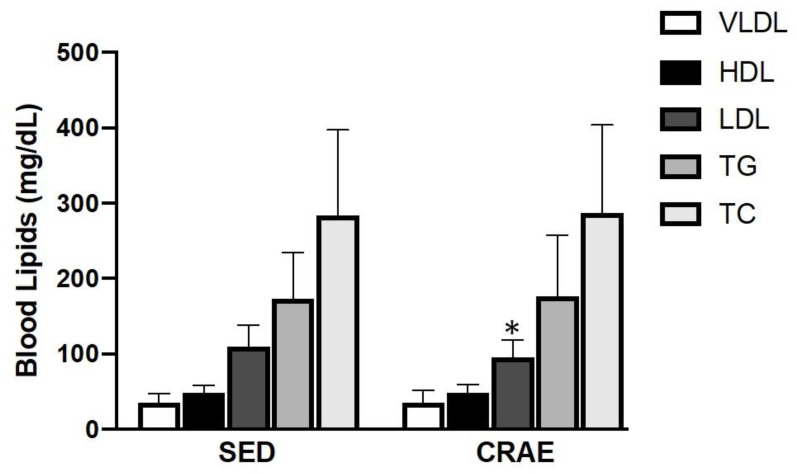
Differences in blood lipids including very low-density lipoprotein (VLDL), high-density lipoprotein (HDL), low-density lipoprotein (LDL), triglycerides (TG), and total cholesterol (TC) between the combined resistance and aerobic exercise training (CRAE) and sedentary (SED) groups. LDL was significantly lower in the CRAE group compared to the SED group (110.9 ± 27.7 vs. 94.9 ± 23.5 mg/dL, SED vs. CRAE, respectively; *p* = 0.023). There were no differences in VLDL (35.1 ± 12.2 vs. 35.4 ± 16.6 mg/dL, SED vs. CRAE, respectively; *p* = 0.37), HDL (48.3 ± 9.8 vs. 47.9 ± 11.7 mg/dL, SED vs. CRAE, respectively; *p* = 0.44), TG (173.0 ± 61.9 vs. 176.9 ± 80.4 mg/dL, SED vs. CRAE, respectively; *p* = 0.39), or TC (284.6 ± 112.5 vs. 287.3 ± 116.9 mg/dL, SED vs. CRAE, respectively; *p* = 0.47). Values are Mean ± SD. * *p* < 0.05 vs. SED.

**Figure 4:**
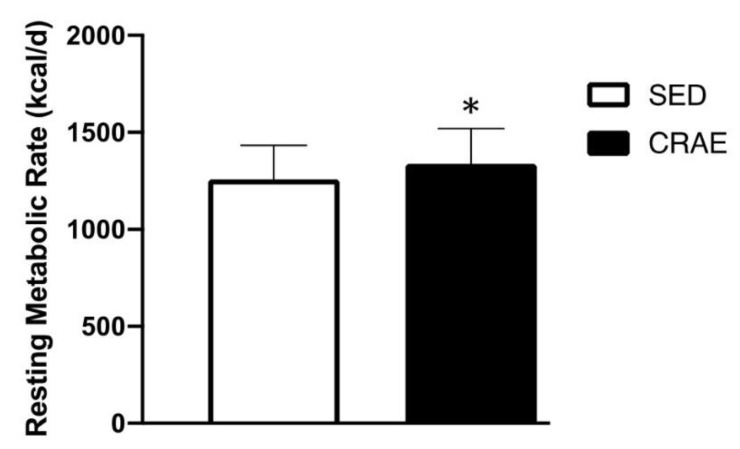
Differences in resting metabolic rate (RMR) between the combined resistance and aerobic exercise training (CRAE) and the sedentary (SED) groups. The CRAE group had significantly greater RMR in kilocalories per day (kcal/d) compared to the SED group (1258 ± 176 vs. 1338 ± 182 kcal/d, SED vs. CRAE, respectively; *p* = 0.038). Values are Mean ± SD. * *p* < 0.05 vs. SED.

**Figure 5:**
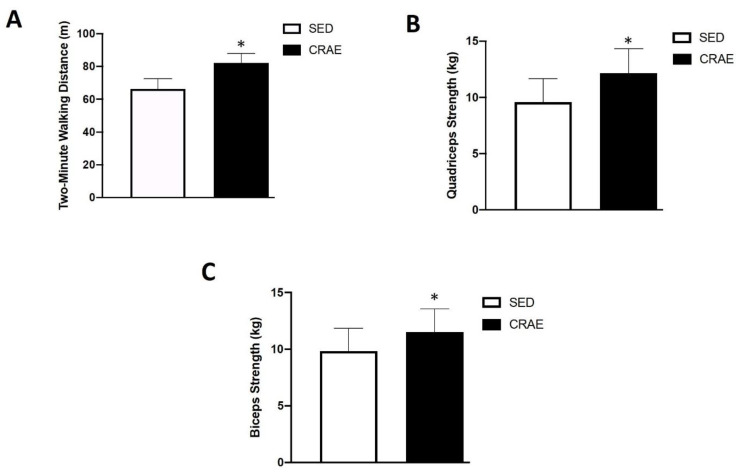
Differences in walking capacity and muscular strength between the combined resistance and aerobic exercise training (CRAE) and sedentary (SED) groups. (**A**) The CRAE group had a significantly greater 2-min walking distance (m) compared to the SED group 66.4 ± 6.1 vs. 82.2 ± 5.7 m, SED vs. CRAE, respectively; *p* = 0.022) (**B**) The CRAE group had significantly greater single-leg extension quadriceps strength (kg) compared to the SED group (9.6 ± 2.0 vs. 12.2 ± 2.0 kg, SED vs. CRAE, respectively; *p* = 0.029) (**C**) The CRAE group had significantly greater two-arm curl biceps strength (kg) compared to the SED group (9.8 ± 2.0 vs. 11.5 ± 2.0 kg, SED vs. CRAE, respectively; *p* = 0.046). Values are Mean ± SD. * *p* < 0.05 vs. SED.

None of the study participants reported any unfavorable symptoms/signs or adverse side effects resulting from their habitual CRAE training or the laboratory tests. Participants were placed in the CRAE training group (CRAE), sedentary control group (SED), or were ineligible for the measurements in this study (Figure 1). Out of the possible 153 participants, 52 were excluded due to partaking in smoking, having psychiatric conditions and/or cognitive dysfunction, having some form of cardiovascular disease, renal disease, thyroid disease, or they declined to participate. The final *n* number was 101. The CRAE group self-reported 80% adherence to the exercise protocols, and average duration of CRAE training participation was 1.0 ± 0.1 years. The CRAE and SED groups had several comorbidities and were on several respective medications for their conditions (Table 2). There were significant differences (*p* < 0.05) for arterial stiffness, systolic BP, LDL, body fat percentage, walking capacity, and muscular strength between the CRAE and SED groups. Compared to the SED group, the CRAE group had significantly lower baPWV (12.8 ± 1.8 vs. 12.1 ± 2.0 m/s, SED vs. CRAE, respectively; *p* = 0.041), systolic BP (137.0 ± 33.0 vs. 120.0 ± 15.0 mmHg, SED vs. CRAE, respectively; *p* = 0.03), LDL (110.9 ± 27.7 vs. 94.9 ± 23.5 mg/dL, SED vs. CRAE, respectively; *p* = 0.023), and body fat percentage (33.0 ± 7.0 vs. 27.0 ± 7.0, SED vs. CRAE, respectively; *p* = 0.028) (Figure 2 and Figure 3, Table 3). The CRAE group had significantly greater RMR (1258 ± 176 vs. 1338 ± 182 kcal/d, SED vs. CRAE, respectively; *p* = 0.038) (Figure 4), 2-min walking distance (66.4 ± 6.1 vs. 82.2 ± 5.7 m, SED vs. CRAE, respectively; *p* = 0.022), quadriceps strength (9.6 ± 2.0 vs. 12.2 ± 2.0 kg, SED vs. CRAE, respectively; *p* = 0.029), and biceps strength (9.8 ± 2.0 vs. 11.5 ± 2.0 kg, SED vs. CRAE, respectively; *p* = 0.046) compared to the SED group (Figure 5). There were no significant differences in diastolic BP (68.0 ± 15.0 vs. 67.7 ± 10.0 mmHg, SED vs. CRAE, respectively; *p* = 0.12), TC (284.6 ± 112.5 vs. 287.3 ± 116.9 mg/dL, SED vs. CRAE, respectively; *p* = 0.47), VLDL (35.1 ± 12.2 vs. 35.4 ± 16.6 mg/dL, SED vs. CRAE, respectively; *p* = 0.37), HDL (48.3 ± 9.8 vs. 47.9 ± 11.7 mg/dL, SED vs. CRAE, respectively; *p* = 0.44), or TG (173.0 ± 61.9 vs. 176.9 ± 80.4 mg/dL, SED vs. CRAE, respectively; *p* = 0.39) between groups (Figure 2 and Figure 3).

## 4. Discussion

The present study was conducted to examine the impacts of habitual CRAE training over 1 year in mid-to-late elderly postmenopausal women compared to a sedentary cohort of similar age. We previously indicated that 12 weeks of CRAE training is a useful exercise modality to reduce risk factors for CVD in postmenopausal women [13,14]. Our current results also support CRAE training in the long-term for reducing risks for metabolic syndrome and improving physical fitness levels. In the present study, we revealed for the first time that habitual CRAE training over the course of 1 year positively influences CVD risk factors such as arterial stiffness, systolic BP, LDL levels, and body fat percentage in elderly postmenopausal women participating in CRAE training compared to their sedentary peers. Additionally, the habitual CRAE training group demonstrated better overall indices of physical fitness, as supported by significantly greater resting energy expenditure, 2-min walking distance, and muscular strength compared to the sedentary group. To our knowledge, this is the first study to demonstrate that habitual CRAE training is specifically beneficial for elderly postmenopausal women to reduce several metabolic syndrome and cardiovascular risks while improving overall physical fitness. This combination of reduced risk factors for metabolic syndrome, and enhanced fitness levels may ultimately contribute to improved quality of life in this population.

### 4.1. Arterial Stiffness and Blood Pressure

Different modes of aerobic exercise training alone for 8–12 weeks (swimming, stair climbing, and all-extremity ergometry) have been shown to significantly decrease arterial stiffness in postmenopausal women and older adults [24,25,26], whereas high-intensity interval training and resistance training for 8–12 weeks showed no differences in arterial stiffness in similar populations [26,27]. Furthermore, CRAE training for 12 weeks has been shown to reduce arterial stiffness in postmenopausal women [13,28]. In the present study, baPWV was significantly lower in the CRAE group versus the SED group by ~1 m/s (Figure 2A). This finding may be clinically beneficial for this population, for it has been previously reported that an increase in baPWV by as little as 1.0 m/s is associated with a 12% increase in risk for cardiovascular event occurrence [29], and baPWV has been suggested as an independent predictor of vascular ageing and CVD development [28]. Therefore, with the CRAE group demonstrating significantly lower PWV compared to the SED group, this may suggest that habitual CRAE training may help with delaying age-associated vascular remodeling.

According to the American Heart Association (AHA), BP greater than or equal to 130/85 mmHg is considered to be a component of metabolic syndrome. We and others have previously shown that CRAE training for 12 weeks (3 times per week) significantly reduces BP in elderly individuals and postmenopausal women with hypertension [14,30]. The present study examined habitual CRAE over 1 year in a similar population, and systolic BP was significantly lower in the CRAE group compared to the SED group (Figure 2B). Importantly, the average systolic BP observed in the CRAE group compared to the SED group (~17 mmHg difference) not only puts postmenopausal women who underwent CRAE training out of the specified BP range for metabolic syndrome, but also has been associated with ~40% lower risk of stroke and ~30% lower risk of ischemic heart disease [31].

Several mechanisms may be partially responsible for the significantly lower baPWV and BP in the CRAE group versus the SED group, including reduced inflammation and improved nitric oxide (NO) bioavailability. Elevated levels of proinflammatory markers, such as c-reactive protein (CRP), are associated with increased prevalence of arterial stiffness, high blood pressure, and CVD development [32]. Yearlong aerobic training was shown to reduce CRP in postmenopausal women, thereby supporting the use of exercise to reduce systemic inflammation and likelihood of disease development [33]. Considering inflammation is associated with arterial stiffness and hypertension, reductions in inflammation may serve as a mechanism for vascular protection. Additionally, we have shown that 12 weeks of CRAE training increases markers of NO bioavailability and reduces arterial stiffness and BP [14]. This may be important for protecting against detrimental vascular remodeling [34] and hypertension development [34,35]. However, these potential mechanisms underlying improved vascular function in the CRAE group require further study.

### 4.2. Blood Lipid Profiles

Following menopause, blood lipid profiles become abnormal, typically including changes such as increases in TC and LDL and decreases in HDL [36]. This rise in TC and LDL is thought to contribute to an increased risk of atherosclerosis and CVD development [37]. Optimal levels for TC, LDL, and HDL are <200 mg/dL, <100 mg/dL, and >40 mg/dL, respectively [38]. It is well-accepted that exercise training can improve blood lipid profiles, therefore using exercise to improve blood lipids in postmenopausal women may help reduce risks for atherosclerosis and CVD.

Wooten and colleagues (2011) reported that 12 weeks of resistance training significantly reduced total cholesterol and LDL levels in postmenopausal women [39]. Additionally, Ammar and colleagues (2015) have shown that total cholesterol and LDL levels decrease through aerobic exercise in postmenopausal women [40]. While there have been numerous studies performed to examine the effects of resistance training and aerobic training separately on blood lipid profiles, there has been limited research examining how CRAE training influences blood lipid profiles, especially in elderly postmenopausal women. In the present study, LDL levels were significantly lower in the CRAE group compared to the SED group (Figure 3). This difference in LDL levels (~15 mg/dL) not only puts CRAE elderly postmenopausal women below the recommended level, but also the degree of LDL lowering is proportional to the relative degree of CVD risk reduction [41].

### 4.3. Body Composition and Resting Metabolic Rate

It has been previously reported that menopause is associated with an increase in total body fat and abdominal obesity [42], and accumulation of total body fat has been shown to be linked to hypertension and dyslipidemia [43,44]. Willis and colleagues (2012) showed that resistance training alone does not reduce body fat as effectively as aerobic training [45], and we have previously demonstrated that 12 weeks of CRAE training is an effective exercise modality to reduce body fat percentage in postmenopausal women [14]. Thus, it may be speculated that habitually CRAE-trained individuals over one year may produce similar results. In the present study, the CRAE group had a significantly lower body fat percentage compared to the SED group (Table 3). Reducing body fat is especially beneficial for older adults due to its associations with hypertension, dyslipidemia, and pulmonary function [43,44,46], and therefore may play a preventative role in CVD development.

Additionally, increased body fat percentage with age is often associated with a loss in energy expenditure [47], and this is often due to reduced physical activity and a sedentary lifestyle [48]. In the present study, the CRAE group demonstrated significantly greater RMR versus SED (Figure 4), which may be due to the physically active versus overall sedentary lifestyle differences. In addition to RMR being significantly greater in the CRAE group, a trend was noted (*p* = 0.052) for greater caloric intake in the CRAE group (Table 3). The present study, however, only examined caloric intake and did not include nutrients and/or minerals.

### 4.4. Walking Capacity and Muscular Strength

Participation in regular exercise and adopting an active lifestyle are promising interventions that promote healthy aging, reduce the likelihood for physical detriments, and improve quality of life in older adults [49]. Although the 6-min walk test is a common test of physical capacity to perform activities of daily living [50], some individuals may lack the ability to walk for a 6-min time period. Therefore, shorter walk tests, such as the 2-min walk test, have been used to estimate physical capacity. Previously, Diehr and colleagues (2010) reported that even a modest increase in walking distance is linked to lower difficulties in activities of daily living [51]. In the present study, the 2-min walk distance was significantly greater in the CRAE group versus the SED group (Figure 5A), suggesting a higher level of walking capacity in the CRAE group. These results indicate that the CRAE group walks ~0.7 m/s while the SED group walks ~0.55 m/s. This reduced walking distance and speed in the SED group may not have only be affected by the sedentary lifestyle, but also by the slightly higher prevalence of arthritis in this group compared to the CRAE group (Table 2). The greater walking speed and distance represented in the CRAE group may be particularly relevant for the performance of activities of daily living in this population, for it has been reported that gait speed is a useful indicator for performance of independent activities of daily living in geriatric populations [52].

Prevalence of sarcopenia, the age-associated natural loss of skeletal muscle mass, is increasing, affecting nearly 20% of postmenopausal women [53]. Additionally, muscular strength is necessary to perform activities of daily living and has been reported to have protective effects against arterial stiffness and CVD development [24,54]. Incorporating resistance training has been thought to be an ideal intervention for improving muscle function and activities of daily living, such as walking capacity and gait speed in older adults [55]. Conceicao et al. (2013) demonstrated that 16 weeks of resistance training can increase lean muscle mass and strength in women [56], and Leite and colleagues (2010) showed similar effects with the benefit of an improved quality of life [57]. Therefore, resistance exercise has been regarded as an effective intervention for increasing lean mass and muscular strength in women, which may help protect against sarcopenia that is associated with postmenopausal phase. The present study demonstrated that the CRAE group had significantly greater quadriceps and biceps strength compared to the SED group (Figure 5B,C). These strength improvements suggest that habitual CRAE training supports total body strength, which may be beneficial for performing activities of daily living in this population [58].

### 4.5. Lifestyle and Medication Use

The benefits of long-term exercise on disease conditions have been studied and can promote improvements in health status while protecting against several chronic diseases such as diabetes, hypertension, osteoporosis, and cardiovascular diseases [59]. The present study supports this notion, which may be evidenced by the medication use between the CRAE and SED groups. For example, a higher percentage of the SED group demonstrated greater prevalence for chronic disease conditions (Diabetes, hypertension, dyslipidemia, and arthritis) and were using a higher percentage of medications for these respective conditions compared to the CRAE group, with NSAIDS being statistically significant (Table 2). This may suggest that the regular exercise participation by the CRAE group may help reduce these disease conditions and/or associated symptoms, which may be beneficial for improving overall quality of life while reducing risks for CVD. However, it may be important for future investigations to note a more comprehensive and detailed medication history (e.g., when medications were started and/or stopped by physician recommendation) to determine the sole effect of exercise as a lifestyle change intervention.

It is also important to consider that other lifestyle factors such as socioeconomic status and education history can impact perceived barriers to exercise participation [60,61]. The majority of our study population had at least finished their high school degree and most had been working as housewives for their entire lives. We unfortunately do not have detailed information about their social roles and socioeconomic status. However, all participants attended local community centers and lived in a very similar economic and social-cultural area to one another, and these participants were mostly considered to be middle-class. Future studies should investigate socioeconomic status and social roles, as this information may help determine potential perceived barriers to exercise participation.

### 4.6. Limitations

Our study has several limitations. First, participants were recruited from local community health centers, and therefore the groups are not precisely age-matched (~3 years difference between groups). Future work should incorporate stricter guidelines for age-matched groups for better controlled comparisons regarding the impacts of regular exercise on health status in similar populations.

Second, this was a comparison-based study and not an interventional study, therefore baseline data were not collected. More investigation is needed to quantify exact physiological changes that may occur pre-to-post habitual CRAE training, and this may be further compared to age-matched counterparts. Additionally, it is important to note that the data we have acquired in this study are comparable to data from other 12-week training studies that we and others have conducted [13,14,30]. In both the 12-week and the 1-year designs, there were reductions in arterial stiffness, SBP, and body fat percentage while there were improvements in muscular strength, therefore 1-year of habitual CRAE training can help maintain these results we see in as little as 12 weeks. For example, the CRAE group and SED groups in the present study demonstrate similar baPWV values when compared to a 12-week CRAE training group and a 12-week sedentary control group in a similar study population: 11.9 m/s vs. 12.1 m/s, 1 year habitual CRAE vs. 12 weeks CRAE training, respectively, and 12.8 m/s vs. 12.7 m/s, 1 year SED group vs. 12 weeks sedentary control group, respectively [13]. Therefore, the habitual CRAE training may help maintain these improvements in vascular adaptations and may delay the aging-associated deterioration in vascular health.

Third, we did not assess estrogen levels between groups. Although we have previously demonstrated that 12 weeks of CRAE training and 12 weeks of resistance band-based training improves levels of estrogen in postmenopausal women [17,18], to our knowledge, it has not been documented how habitual CRAE training may impact estrogen levels in postmenopausal populations. Estrogen levels may be important to assess this in future studies of this type, since estrogen deficiency is thought to contribute to vascular dysfunction and hypertension in this population [9,12].

Last, we did not screen for other underlying health conditions such as osteoporosis or osteopenia. A more comprehensive screening of health history and underlying conditions may be necessary for future studies. Additionally, participants were excluded if they were participating in hormone replacement therapy due to the confounding effects it may have on the aging vasculature in postmenopausal women. This was done to determine the ability of habitual exercise alone to improve vascular function and metabolic profiles, however since hormone replacement therapy is common among postmenopausal women and these effects in addition to exercise training warrant further investigation. We also did not record whether menopause occurred naturally or surgically. It has been well documented that following menopause, vascular ageing and dysfunction are accelerated [9,62], and that menopause occurring later in life is associated with reduced CVD risk [63]. Therefore, the type of menopause (natural or surgical) likely does not affect associations between age at menopause onset and mortality rate [64], and this is likely also the case in the present study.

## 5. Conclusions

As the aging population continues to increase, the prevalence of the postmenopausal population will concomitantly increase. As a result, the need for maintaining optimal metabolic parameters, vascular function, and physical fitness in postmenopausal women is crucial to not only minimize risks for metabolic syndrome and CVD but also for the quality of life in this population. Our results indicate that the use of habitual CRAE training can be a beneficial lifestyle intervention to protect against CVD risks while promoting physical fitness in mid-to-late elderly postmenopausal women.

## Figures and Tables

**Table 1:** Combined resistance and aerobic exercise (CRAE) training program.

Time Frame	Order	Exercise	Duration	Sets/Repetitions or Intensity	Frequency
≥1 year	Warm-up	Static stretching	5 min		3x/week
		Walking			
	Resistance training	Pushup	20 min	3 sets, 10–15 reps; RPE 12–14	
		Seated row			
		Shoulder flexion			
		Elbow flexion/extension			
		Squat			
		Leg press			
		Calf raise			
		Hip flexion/extension			
	Aerobic training	Walking and jogging	30 min	50–60% HRR; RPE 12–14	
		Cycling			
	Cool-down	Static stretching	5 min		
		Walking			

HRR: heart rate reserve; RPE: rating of perceived exertion.

**Table 2:** Participant comorbidities and medications for the sedentary (SED) and combined resistance and aerobic exercise (CRAE) groups (*n* and % prevalence in each group) * *p* < 0.05 vs. SED.

Comorbidity or Condition	SED (*n* = 44)		CRAE (*n* = 57)		*p*-Value
Diabetes mellitus	6	14%	4	7%	0.527
Hypertension	23	52%	26	46%	0.668
Dyslipidemia	16	36%	15	26%	0.857
Arthritis	22	50%	21	37%	0.879
**Medications**					
Angiotensin-converting enzyme inhibitors	11	25%	7	12%	0.346
Diabetic medication/insulin therapy	6	14%	4	7%	0.527
Beta blockers	8	18%	14	25%	0.201
Calcium channel blockers	4	9%	5	9%	0.739
Non-steroidal anti-inflammatory medication	19	43%	12 *	21%	0.047

**Table 3:** Participant characteristics in the sedentary (SED) and combined resistance and aerobic exercise (CRAE) groups.

Total Participants (*n* = 101)							
	SED (*n* = 44)			CRAE (*n* = 57)			*p*-Value
Age, y	78.0	±	7.0	75.0	±	6.0 *	0.027
Mass, kg	57.0	±	9.0	56.0	±	10.0	0.100
Height, cm	160.0	±	1.0	150.0	±	1.0 *	0.032
BMI, kg/m^2^	25.0	±	3.0	23.0	±	4.0 *	0.047
Body fat, %	33.0	±	7.0	27.0	±	7.0 *	0.028
Postmenopausal duration, y	25.0	±	11.0	23.0	±	12.0	0.330
Dietary intake, kcal/d	1350.0	±	143.0	1372.0	±	156.0	0.052

Values are Mean ± SD. SED: sedentary control group, CRAE: combined resistance and aerobic exercise training group. BMI: body mass index. Values are Mean ± SD. * *p* < 0.05 vs. SED
